# Systematic Assessment of Health Risk from Metals in Surface Sediment of the Xiangjiang River, China

**DOI:** 10.3390/ijerph17051677

**Published:** 2020-03-04

**Authors:** Huan Li, Liyuan Chai, Zhihui Yang, Weichun Yang, Qi Liao, Zhe Cao, Yanchun Peng

**Affiliations:** 1 Institute of Environmental Science and Engineering, School of Metallurgy and Environment, Central South University, Changsha 410083, China; lhcsu22@163.com (H.L.); lychai@csu.edu.cn (L.C.); yangzh@csu.edu.cn (Z.Y.); yang220@csu.edu.cn (W.Y.); 2 Changsha Environmental Protection College, Changsha 410004, China; hgcaozhe@126.com (Z.C.); lspring0109@163.com (Y.P.); 3 Chinese National Engineering Research Center for Control & Treatment of Heavy Metal Pollution, Changsha 410083, China

**Keywords:** screening evaluation, sediment, heavy metal, health risk, Xiangjiang River

## Abstract

The common empirical screening method is limited to a preliminary screen target from vast elements for human health risk assessments. Here, an element screening procedure was developed for assessing the human health risk of the elements in the sediment of the Xiangjiang River. Ninety-six surface sediment samples from eight sampling stations were collected and 27 elements of each sample were investigated. Thirteen of the 27 elements were screened for human health risk assessments through the three-run selections by calculating anthropogenic factors, building element maps, and the removal of unnecessary elements. Pb posed the greatest health risk and exhibited a potential noncarcinogenic risk for adults at the stations S4 and S5, although no visible noncarcinogenic and carcinogenic risk for adults and children in the Xiangjiang River. Our study also suggested that the chalcophile elements were associated with greater health risk, compared to the lithophile and siderophile ones.

## 1. Introduction

Sediment plays a significant role in the transport and storage of hazardous metals [1,2,3,4,5]. As a consequence of increasing human activities, contamination especially by metals in sediment has become a severe problem [6]. Not only do these pollutants cause aquatic ecosystem disturbance; but some of them (Cd, Pb, Hg, Zn, Cu, As and Co, etc.) subsequently enter the food chain, and threaten human health by poisoning and accumulating in the aquatic and benthic microorganisms and fishes, etc. [7,8,9,10,11,12].

In recent years, the health risk from heavy metals in sediment and soil has been extensively studied by multiple methods [13,14,15,16]. Previous studies mainly focused on contamination status, tracing contamination source and human health risk assessment [2,6,16,17,18,19]. However, there are two critical questions limited in the aforementioned methods: (a) What is the relationship between some elements (especially unattended elements B, Mg, Nb, Na, Ca, Zr, Ba, K, Be, Sc and Y, etc.) with mutually independent characteristics and health risk? (b) How to effectively screen target elements from vast metals for health risk assessments in a specific polluted area?

Although the elements are screened for the health risk assessments in the previous studies, their core principles are empirical [14,20,21,22,23,24,25,26]. The five toxic heavy metals (Hg, Cd, As, Pb, Cr) or other characteristic metals (Ag, Cu, Zn, Co, Mn, V, Ni, Se and Cs, etc.) were considered as the objects for regional health risk assessments [3,8,12,16,26,27,28,29,30,31,32,33]. To our knowledge, there is no hierarchical screening method for preliminary screening in the risk assessment processes.

The Xiangjiang River, which is the largest drainage system in Hunan province, China, has been receiving metals from the mining and smelting of nonferrous metals for hundreds of years. However, the health risk assessments for the sediment of the Xiangjiang River were disturbed by the complex relationships of vast metals. Moreover, some unattended metals, especially Be, Co, Ti, V, Sc and Mg, were rarely concerned in the health risk assessment. The objectives of this study were: (1) to develop a framework to preliminarily screen the prior evaluation factors which should be included in the risk assessments; and (2) to investigate human health risk of the selected metals in the Xiangjiang river. Our study will potentially provide a reference for policy decision on accurately confirming regional pollutants.

## 2. Materials and methods

### 2.1. Study Area

The Xiangjiang River, a tributary of the Yangtze River, originates from Haiyang Mountain, Guangxi, and flows through eight cities in in Hunan province. Its basin is a bicarbonate type geological structure and physiognomy [34]. It drains an area of approximately 94,660 km^2^, and has a total length of approximately 856 km (670 km in Hunan) [35,36]. The vegetation is dense, with intensive biological activities, because of the relatively warm and humid climate. The main type of soil in the drainage basin is red soil, which is relatively rich in the elements Al, Fe and Ti. 

### 2.2. Sediment Sampling

**Figure 1:**
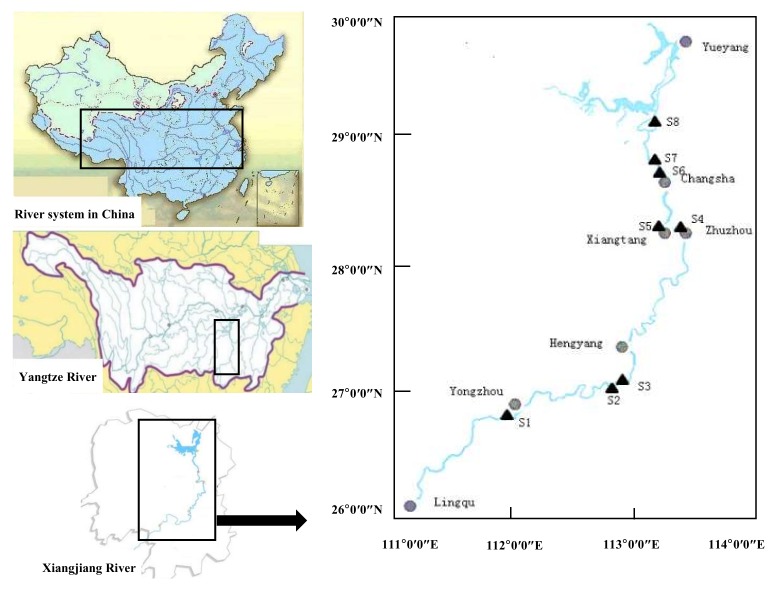
Location of sampling sites in the Xiangjiang River, China.

Surface sediment samples were collected from eight stations (Table 1) in the Xiangjiang River basin (Figure 1). Three sampling sites were set in each station: both sides and middle of the river. Four surface sediment (depth: 0–15 cm) samples for each sampling site were collected by a grab sampler (ZYC-200B, Hangzhou Yijie Technology Co. Ltd., Hangzhou, China) and stored in plastic bags prior to shipping to our laboratories. 

### 2.3. Chemical Analyses of the Sediment Samples

Elements in the samples were determined following the published protocols [37,38,39]. Each sediment sample was dried and passed through a 150-μm nylon sieve, and then 40 mg of the sample was digested with 1 ml of HNO_3_ and 3 ml of HF at 130 °C for 72 h in a Teflon shaker. After cooling, the solution was heated to 120 °C for 12 h after adding 0.5 ml of HClO_4_ until the white smoke was gone. The residue was dissolved with 1 ml of HClO_4_ and 1 ml of deionized water in a sealed vessel for 12 h, and added to 40 ml with deionized water. The concretions of Zn, Pb, Cd, B, Mn, Mg, V, Al, Nb, S, P, Ni, Co, Fe, Cr, Na, Be, Ca, Cu, La, Sc, K, Ti, Zr, Y and Ba were simultaneously determined with an inductively coupled plasma mass spectrometry (ICP-MS, Agilent 7500 series, USA). Each sediment sample (300 mg) also was digested with aqua regia (10 ml) at 95 °C for 2 h, followed by adding 5 ml of HCl, 5 ml of thiourea and aqua regia to 50 ml to determine the As concentration by using an atomic fluorescence spectrophotometry (AFS-810, Beijing Titan Instrument Corp., Beijing, China). The average concentration of an element in the four samples of a sampling site was considered as the concentration of the metal in the site, and was used for the further analyses.

Standard laboratory operating procedures and calibrations were used for quality assurance. China Stream Sediment Reference Materials GBW07309 (GSD-9) and GBW07311 (GSD-11) were used as quality control. The efficiency of recovery for the metals from the standard materials was 91%–105%. In addition, each analysis was carried out in triplicate, and the standard deviation (SD) was within ± 5% of the mean value.

### 2.4. Human Health Risk Assessment Screening Procedure for Sediments of the Xiangjiang River

#### 2.4.1. Overview of the Procedure

**Figure 2:**
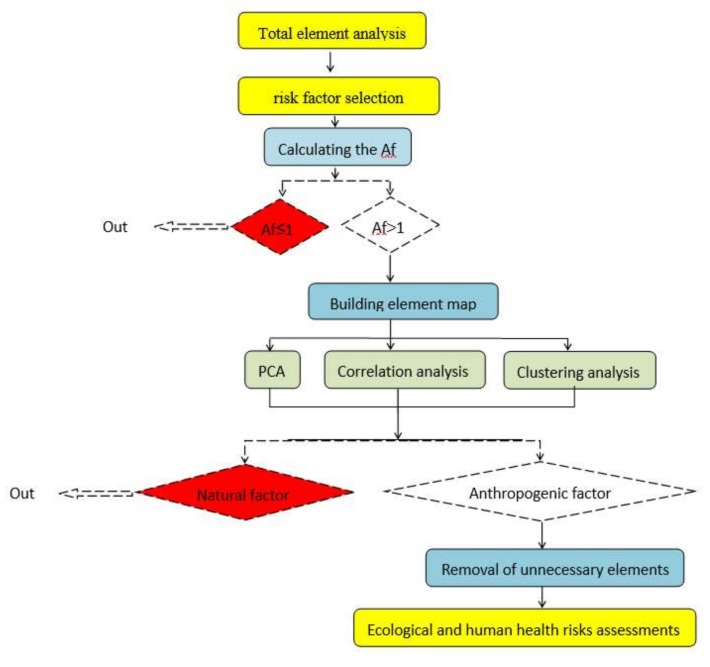
Framework for the health risk assessment procedure.

A human health risk assessment screening procedure (HRASP) was used to distinguish the contribution of an element in sediment on human risk for the Xiangjiang River. The framework of HRASP is shown in Figure 2. There are three screening runs before ecological and health risk assessment. 

#### 2.4.2. First Step: Anthropogenic Factor (Af)

The anthropogenic factor (Af) is set as the first selection run, due to its simplicity and clarity, which is a presumed approach to estimate the anthropogenic impacts upon sediment. The Af is calculated using the following formula [40]:

Af = *Mo/Ms*, (1)

where Ms and Mo are the mean concentrations of an element in the headwater area and sampling sites of the Xiangjiang River. The classifications of the Af index for an element are as follows: 100% × Af ≤ 1: no anthropogenic impacts; 80% × Af ≤ 1: low anthropogenic impacts; 80% × Af > 1 anthropogenic impacts.

#### 2.4.3. Second Step: Build Element Map

There are three methods to build the element map. The principal component analysis (PCA), correlation analysis and cluster analysis were used investigate the sources of elements in sediment samples. All statistical analyses were performed with the SPSS 19.0 statistical program (IBM, Inc., Armonk, NY, USA). 

Correlation analyses were performed to determine the relationships between elements. The PCA of the annual mean of elements with the varimax rotation of the standardized component loadings was conducted by the eigenvalue decomposition. The Kaiser–Meyer–Olkin (KMO) and Bartlett’s tests were performed to evaluate the validity of the PCA, and the principal component value > 1 was retained until the cumulative variance was > 80%. Standardized data sets were subjected to cluster analysis using the Minkowski and Ward method [41,42], and depicting relationships among elements with a dendrogram. Pearson correlation was also used to explore relationships among the elements.

#### 2.4.4. Human Health Risk Assessment

The human health risk assessments of the selected elements in sediment were referred to the soil assessment methods by USEPA [43], although exposures to sediment differed from that to soil. The chronic daily intake (CDI) and hazard quotient (HQ) index were used to evaluate the noncarcinogenic risk for residents, while CDI and carcinogenic risk (CR) were used to evaluate the carcinogenic risk. The pathways of pollutant entry into the human body include ingestion, dermal contact and inhalation. The following formulas were used to calculate carcinogenic and noncarcinogenic risk. All terms are defined with units in Table 2.

(2) $$CDI\left(ingestion\right)=\frac{C\times IRS\times ED\times EF\times CF}{BW\times AT}$$

(3) $$CDI\left(dermal\right)=\frac{C\times SA\times AF\times ABS\times EF\times ED\times CF}{BW\times AT}$$

(4) $$CDI\left(inhalation\right)=\frac{C\times ET\times EF\times ED}{PEF\times 24\times AT}$$

(5) $$HQ_{i}=\frac{CDI_{i}}{RfD_{i}}$$

(6) $$HI=\sum_{i=1}^{n}HQ_{i}$$

(7) CR=CDI×SF

If the HI value is less than 1.0, the exposed individual is unlikely to experience obvious adverse health effects. Otherwise, when HI values exceed 1.0, noncarcinogenic risk may occur [44,45].

When CR is between 1.0 × 10^–4^ and 1.0 × 10^–6^, it is generally considered an acceptable cancer risk, but risk values exceeding 1.0 × 10^–4^ are considered a carcinogenic risk to the human body [46].

**Table 2:** The formula explanations and value sources of human health risk assessment.

Name	Full name	Value	Unit	Reference
CDI	Chronic Daily Intake	Value of calculation	μg/kg bw/day	[45]
C	The concentration of toxic elements in the sediment	measured value	mg/kg	[45]
IRS	Ingestion rate	114	mg/day	[44]
ED	Exposure duration	35 for adult 6 for children	year	[45]
EF	Exposure frequency	350	days/year	[45]
BW	Body weight	70 for adult; 15 for children	kg	[45]
AT	Average time	ED × 365 for non-carcinogens; 25,550 for carcinogens	days	[45]
SA	Exposed skin surface area	6032 for adult; 2373 for children	cm^2^	[45]
AF	Adherence factor from sediment to skin	0.07 for adult; 0.2 for children	mg/cm^2^	[45]
ABS	Dermal absorption from sediment	0.001	unitless	[45]
CF	The unit conversion factor	10^–6^	kg/mg	[45]
ET	Exposure Time	12	h/day^–1^	[45]
PEF	Particle emission factor	1.36 × 10^9^	m^3^/kg	[45]
HQ	Hazard quotient	Value of calculation	unitless	[44]
RfD	Reference dose of the i-th potentially toxic element	Table 3	mg/kg^−day^	[44,46]
HI	Hazard index	Value of calculation	unitless	[44]
CR	Carcinogenic risk	Value of calculation	unitless	[45,46]
SF	Carcinogenicity slope factor	Table 3	per mg/kg^–day^	[45]

## 3. Results and Discussion

### 3.1. Preliminary Screening of Risk Assessment Factors

#### 3.1.1. Element Characteristics in the Sediment Samples

**Figure 3:**
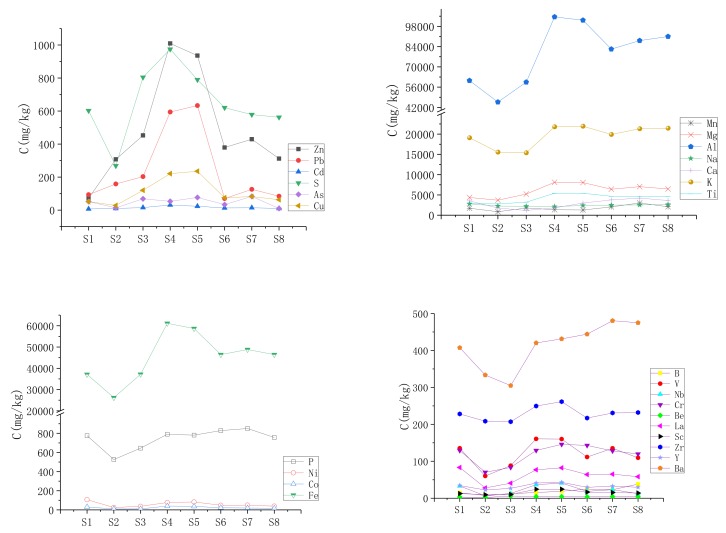
Distribution of elements in the study areas.

The rank of the metals in the sediment samples was in a decreasing order as follows: Al > Fe > K > Mg > Ti > Ca > Na > Mn > P > S > Zn > Ba > Pb > Zr > Cu > V > Cr > La > Ni > As > Y > Nb > Sc > B > Be > Co > Cd (Figure 3). It is better to comprehend the variations within river environments, recognizing the mutual source and relationship correlation among various elements [47]. 

The elements were grouped into four categories: lithophile (B, Mn, Al, Mg, V, Nb, Na, Ca, Zr, Cr, Ba, Ti, K, Be, Sc and Y), chalcophile (Zn, Pb, Cd, As, S and Cu), siderophile (Fe, P, Ni and Co) and La elements through their different chemical properties and distribution in the atmosphere, hydrosphere, lithosphere and biosphere [48].. The concentrations of siderophile, chalcophile and some lithophile elements (Mn, Al, Mg, V, Nb, Zr, Cr, Ba, Ti, K, Be, Sc and Y) increased from the upstream to the midstream (S3, S4, S5), and then decreased in the downstream of the Xiangjiang River. But the contents of the lithophile elements of Na, Ca and Ba, and siderophile element P, slowly increased with the flow direction of the river. The source of an element cannot be traced by its content alone. A study suggested that the siderophile elements are abundant in meteoritic basalts, which cannot be explained by a single-step partial-melting process from a chondritic, metal-containing source [49].

In the present study, the chalcophile elements of Pb, Zn, Cd, As and Cu were abnormally rich in the midstream of the Xiangjiang River. The metals were likely discharged from adjoining mining operations, smelting plants and residences [41,50,51]. The lithophile elements were accumulated in sediment and combined with the diverse metal organic complex or iron oxide forms [52,53]. Meanwhile, other elements (e.g., Mg, Ca and Al) were leached from rocks and soil in a humid climate and fluvial environment. This was similar to other research, such as in the basalts and basanites of the French Massif Central [54]. 

#### 3.1.2. The First Selection-Anthropogenic Factor (Af) 

**Figure 4:**
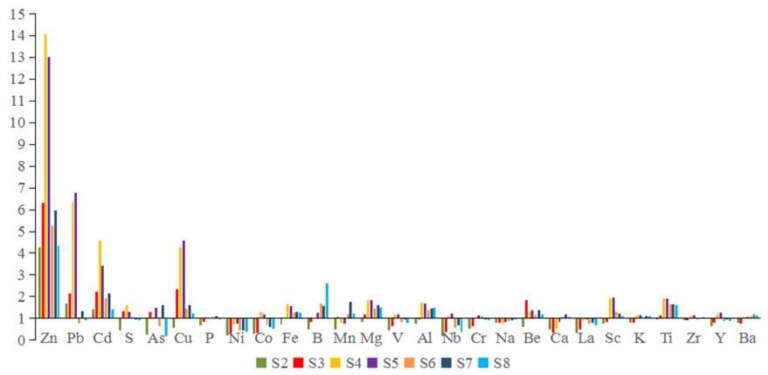
The Af values of the metals in the sediment samples.

Figure 4 shows the Af values of all of the heavy metals at different stations. For the whole river, the Af of As, Cd, Cu, Pb and Zn declined after the sampling stations S4 and S5. The average Af values of the metals in the sediment samples followed the order: Zn > Pb > Cd > Cu > Ti > Mg > Al > Sc > Be > Fe > Mn (Figure 4). Ni, La and Na were excluded from the further human health assessments because of their lower Af values. However, the maximum concentrations of Zn and Pb were about 14 and 7 times higher than their anthropogenic source values, respectively. It indicated that Zn and Pb were mainly from the anthropogenic sources, such as the mining and smelting of metals. 

#### 3.1.3. The Second Selection-Building Element Map

##### Principal Component and Clustering Analyses

**Figure 5:**
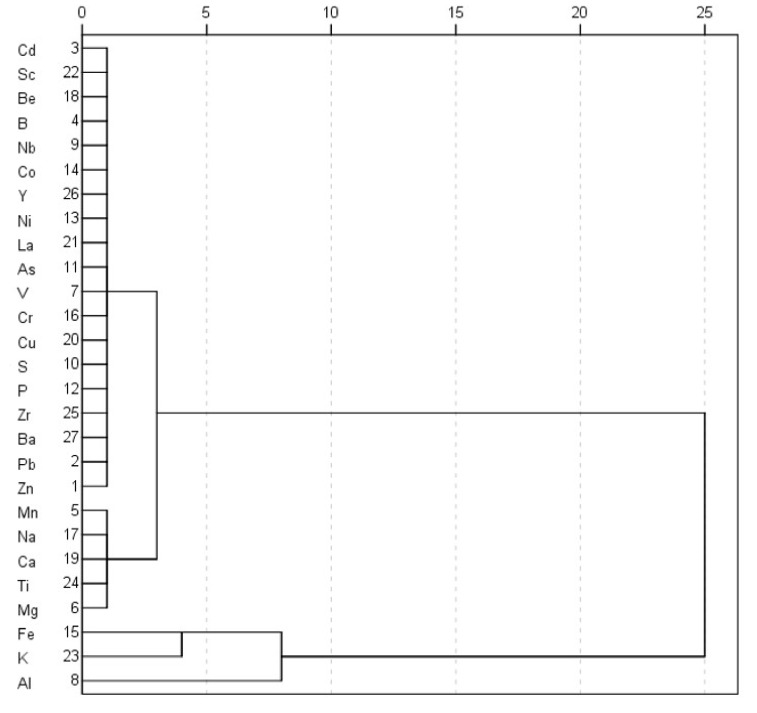
Clustering analyses of elements at all sites.

Both principal component and clustering analyses differentiated the samples according to the sources of their elements. The elements were correlated with the five principal components in the PCA results (Table 4), which were grouped into three clusters by HCA (Figure 5).

The results of PCA indicated that two principal components (PCs) explained 66.7% of the total variance in the data set (Table 4), and allowed the tentative exploration of element sources. The first PCA factor (Table 4) explained 52.89% of the data variance, and was found to have more members of significant variables than the others, and comprising of Zn, Pb, Cd, Mg, V, Al, Nb, S, As, P, Ni, Co, Fe, Cr, Be, Cu, La, Sc, K, Ti, Zr and Y, which correlated with the first cluster in HCA (except Fe, K and Al). This cluster had two sub groups (I: Zn, Pb, Cd, Cu and As; II: Mg, V, Al, Nb, S, P, Ni, Co, Fe, Cr, Be, La, Sc, K, Ti, Zr and Y). The sub-group I was possibly attributed to the intrusion of Pb–Zn mining and smelting industries [41,55,56]. Samples from sites S3 to S5 contributed to this factor. The major Pb–Zn smelting plants and chemical plants were distributed over these areas. 

The second PCA factor with 13.79% of the variance was comprised of B, Ca and Ba, with high loadings, and Na with a relatively low loading. The HCA cluster in Figure 5 showed resemblance of this factor (Ca and Na). Given that B, Ca, Na and Ba were primarily derived from the biogenic carbonates [57,58,59], it was safe to infer that the major source of B, Ca, Na and Ba in the Xiangjiang River was the natural detritus input.

##### Correlation Analysis 

**Figure 6:**
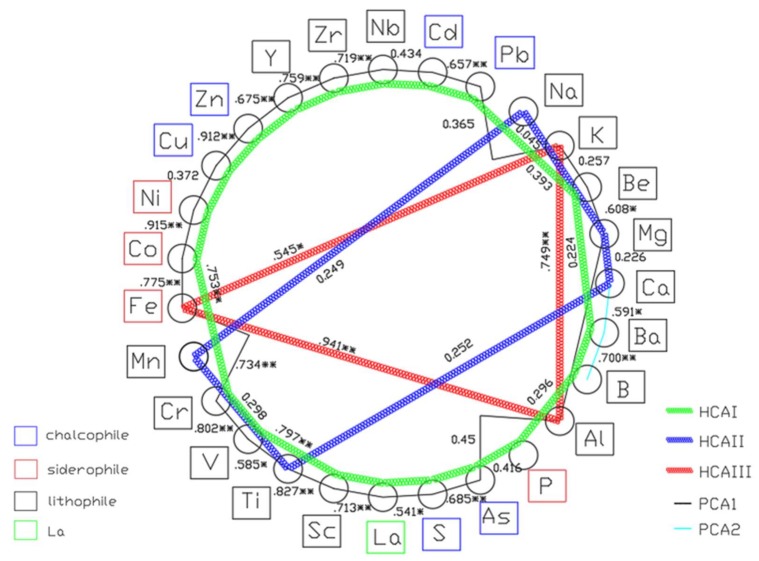
Element map. ** Significant correlation at the 0.01 level (two tailed); * significant correlation at the 0.05 level (two tailed).

There were complicated relationships among elements in the sediment samples due to uncertainties factors [1,6]. An element map (Figure 6) was built according to the periodic table, principal component analysis, cluster analysis and correlation indices, which can help sort out the relationships between the elements. In the element map, all elements were arranged in a circle according to the periodic table. The two lines represented the results of principal component analysis and cluster analysis, respectively, and the number between the lines represented the correlation coefficient of the two elements. As shown in Figure 6, the elements of Zn, Pb, Cu, Cd, Y, Zr and Nb had significantly positive correlation with each other (*p* < 0.01). In addition, there was no significantly negative correlation with other elements. Correlation analysis and Af values suggested that Y, Zr and Nb were derived from soil erosion in the upper reaches of the river, while Zn, Pb, Cu and Cd originated from the anthropogenic activities to discharge the combined industry pollutants with highly complex human-induced and environmental processes in the Xiangjiang River [41,60]. A positive correlation (*p* < 0.05) was presented between the same principal component elements, except the pairs of Ni and Cu, Nb and Cd, P and As and Be and Ba. The pairs of Ni and Cu, Nb and Cd and P and As, belonged to a different geochemical element classification (Figure 6). Through the PCA, the pair of Be and Ba did not belong to the same principal component, and Be possibly derived from the anthropogenic sources, whereas Ba from the biogenic carbonates.

Most of the lithophile elements did not significantly correlate with the chalcophile elements except Sc, Ti, Mg and Al. Each element was associated with at least four other elements (*p* < 0.05), except Na and Ca. Only the pairs of Na and K, Ca and Cr, Ca and Ba were significantly correlated at the *p* < 0.05 level. The siderophile elements were positively correlated (*p* < 0.05). In constructing the element map in the order of the periodic table, a significant correlation (*p* < 0.01) was found among Fe and adjacent elements (elements in group B in the periodic table), except for the pair of Fe and Mn. Not following the explanation for the lack of correlation between Fe and Mn, and it might be the associated mineral combined with the input pollutions. 

Therefore, B, Ca, Na, Ba, Fe, K, Al, Y, Zr and Nb were excluded due to their natural sources for the sediment samples in the Xiangjiang River.

#### 3.1.4. The Third Selection-removal of Unnecessary Elements

Nb is among the lithophile elements with lower concentration in the surface sediment samples of the Xiangjiang River, and was selected out at the step of the element map. S and P are the chalcophile and siderophile elements, respectively, and are not toxic, so their human health risk assessments are unnecessary. Therefore, 13 elements, namely Zn, Pb, Cd, Mn, Mg, V, As, Co, Cr, Be, Cu, Sc and Ti, were included human health risk assessments in the present study.

Each screening step requires that the index value of a pollutant meet the requirements before the next screening step. This procedure provides an efficient and cost-effective assessment of risks to human health in contaminated areas. It results in the more accurate determination of elements posing potential health risks, especially in sensitive locations or conservation areas. It can help in decision-making for policy and remediation management.

### 3.2. Non-carcinogenic Risk in the Sediment Samples of the Xiangjiang River

**Figure 7:**
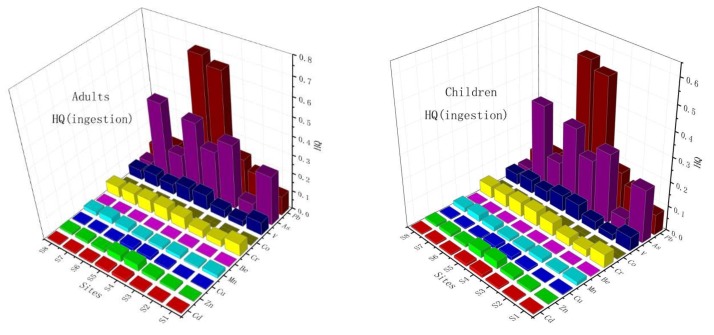

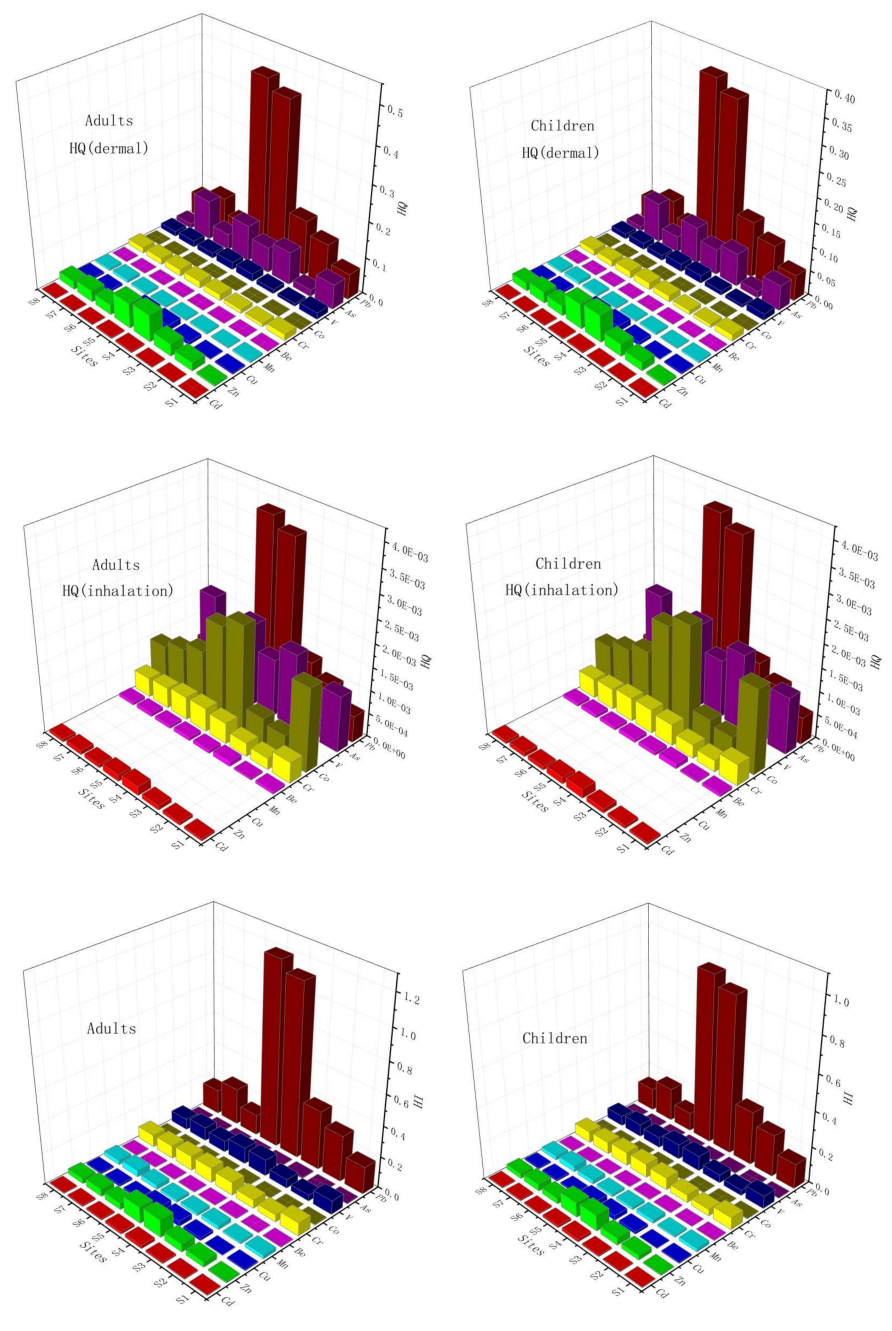

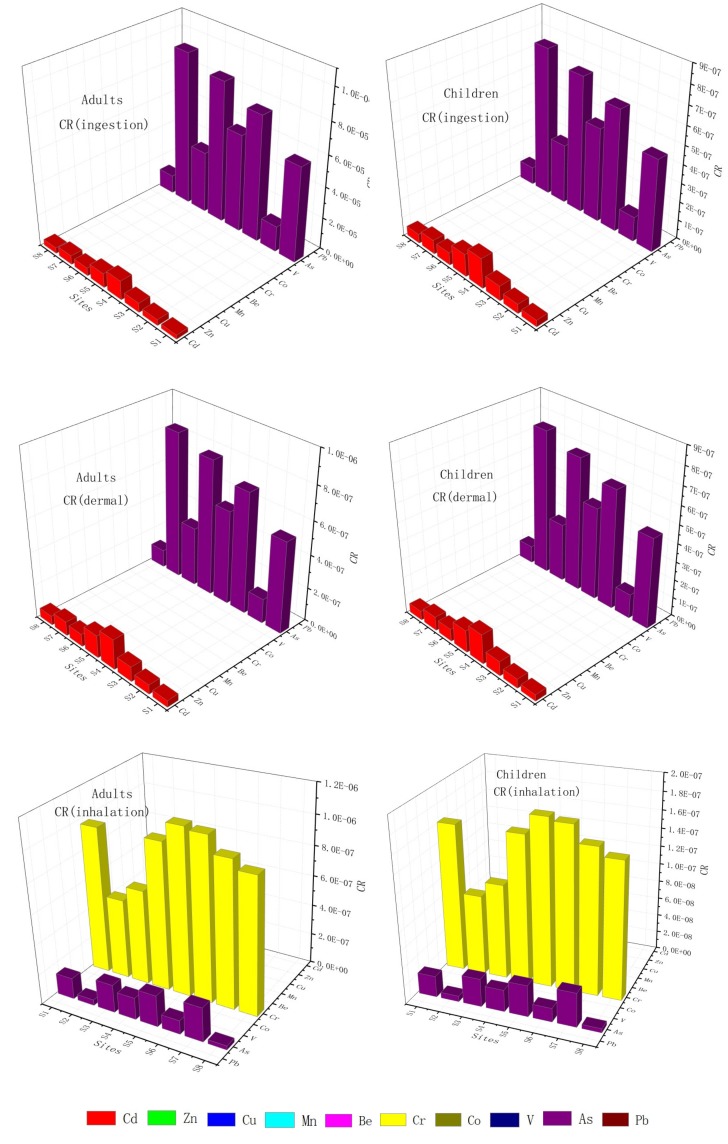
The hazard quotient (HQ), hazard index (HI) and carcinogenic risk (CR) with the study area in sediment.

The results of the hazard quotient (HQ) and chronic hazard index (HI) for the sediment samples of the Xiangjiang River are summarized in Figure 7 with corresponding ingestion, dermal and inhalation reference dose (RfD). The calculated HQ values for the selected elements ranged from 3.59 × 10^–5^ to 7.1 × 10^–1^. The HI values for both children and adults followed the order: Pb > As > Cr > V > Zn > Mn > Cu > Cd > Be > Co. Pb showed a higher noncarcinogenic risk than other elements because of its high concentration and low RfD values [61]. Especially, Cd had a high ecological risk values (Chai et al., 2017), but a low noncarcinogenic risk values, due to its low concentration in the sediment samples or a lower background value. For the Xiangjiang River, the average HI values for adults were higher than for children due to a large dermal contact area. Pb at stations S4 and S5 posed potential noncarcinogenic risk for children and adults, although the HI values of other metals were lower than 1. In conclusion, the noncarcinogenic risk of the chalcophile elements and some of the lithophile elements (V, Mn and Be) were significantly higher than the siderophile elements. 

### 3.3. Carcinogenic Risk in the Sediment Samples of the Xiangjiang River 

The carcinogenic risk (CR) levels of Cd, As and Cr are presented in Figure 7. the carcinogenic risk of As of the three pathways, Cd of ingestion and dermal, Cr of inhalation for adults, were higher than for children. The highest HI and CR values were appeared in the station S4. 

The results of cancer risk assessments were less than 1.0 × 10^–4^, indicating that there is no obvious carcinogenic risk of these heavy metal exposures.

## 4. Conclusions

This study developed a novel methodology regarding the element selecting steps for human health risk assessments. There are three-run selections: calculating anthropogenic factors, building element maps, and the removal of unnecessary elements. Ninety-six surface sediment samples from eight sampling stations were collected, and 27 elements of each sample were investigated. Thirteen of the 27 elements were screened for the risk assessment through the human health risk assessment screening procedure (HRASP). The results showed that the HI values for both children and adults followed the order: Pb > As > Cr > V > Zn > Mn > Cu > Cd > Be > Co, and there is no apparent noncarcinogenic and carcinogenic risk in the Xiangjiang River, except Pb for adults at S4 and S5. The chalcophile elements were shown a greater noncarcinogenic risk than other elements in the sediment samples from the Xiangjiang River. This suggested that the chalcophile elements should be paid more attention in the health risk assessment.

## Figures and Tables

**Table 1:** Geographic background of the sampling stations of the Xiangjiang River.

Station No.	Names of Station	Location	Characteristic
S1	Lvbutou	upstream	the starting point of the Xiangjiang River in Hunan province
S2	Songbo (upstream)	upper and middle reaches	a heavily industrialized region
S3	Songbo (downstream)	upper and middle reaches	a heavily industrialized region
S4	Xiaowang Port	midstream	the most polluted tributary of the Xiangjiang River
S5	Xiangtang	middle and lower reaches	an old industrial region
S6	Changsha	middle and lower reaches	the most populous city
S7	Wangcheng	downstream	downstream of Changsha city
S8	Xiangyin	downstream	the place that ran into Dongting lake

**Table 3:** Summary of reference dose (RfD) and SF values of the selected elements.

Index	Cd	Pb	Zn	Cu	Mn	As	Be	Cr	Co	Ti	V	Sc	Mg
RfD (ingestion) mg·kg^–1^·day^–1^	3.00 × 10^–3^	1.40 × 10^–3^	4.00 × 10^–2^	2.00 × 10^–2^	1.40 × 10^–1^	3.00 × 10^–4^	2.00 × 10^–3^	3.00 × 10^–3^	3.00 × 10^–2^	NA	3.00 × 10^–3^	NA	NA
RfD (dermal) mg·kg^–1^·day^–1^	3.00 × 10^–3^	5.24 × 10^–4^	5.40 × 10^–3^	5.40 × 10^–3^	1.40 × 10^–1^	3.00 × 10^–4^	2.00 × 10^–3^	3.00 × 10^–3^	3.00 × 10^–2^	NA	3.00 × 10^–3^	NA	NA
RfD (inhalation) mg·kg^–1^·day^–1^	5.71 × 10^–5^	5.71 × 10^–5^	NA	NA	NA	1.50 × 10^–5^	2.00 × 10^–5^	1.00 × 10^–4^	6.00 × 10^–6^	NA	NA	NA	NA
SF (ingestion) mg·kg^–1^·day^–1^	5.01 × 10^–1^	NA	NA	1.70	NA	1.50	NA	NA	NA	NA	NA	NA	NA
SF (dermal) mg·kg^–1^·day^–1^	2.00 × 10^1^	NA	NA	4.25	NA	3.66	NA	NA	NA	NA	NA	NA	NA
SF (inhalation) mg·kg^–1^·day^–1^	NA	NA	NA	NA	NA	1.51×10^1^	NA	4.20×10^1^	NA	NA	NA	NA	NA

NA: not applicable.

**Table 4:** Loadings of the elements on Principal Component (PCs) in sediment.

Elements	PC1	PC2	PC3	PC4	PC5
Zn	0.743	–0.377	0.49	–0.056	–0.115
Pb	0.664	–0.513	0.377	–0.339	0.121
Cd	0.602	–0.415	0.31	0.118	–0.318
B	0.338	0.709	0.036	0.079	–0.419
Mn	0.298	0.531	0.098	0.705	0.166
Mg	0.881	0.146	0.419	0.112	–0.062
V	0.916	–0.023	–0.369	0.027	0.03
Al	0.905	0.249	0.277	0.019	–0.167
Nb	0.859	–0.174	–0.354	–0.209	0.195
S	0.719	–0.336	–0.115	0.507	–0.016
As	0.687	–0.284	–0.09	0.335	0.462
P	0.615	0.279	–0.364	0.325	–0.055
Ni	0.655	–0.133	–0.689	–0.103	0.097
Co	0.825	–0.12	–0.462	–0.159	–0.098
Fe	0.972	0.038	0.044	0.103	–0.173
Cr	0.763	0.311	–0.373	–0.101	–0.109
Na	–0.156	0.574	0.393	–0.224	0.559
Be	0.563	–0.21	0.23	0.615	0.265
Ca	0.291	0.711	–0.289	–0.191	0.162
Cu	0.808	–0.424	0.313	–0.069	0.086
La	0.781	0.054	–0.506	–0.119	0.082
Sc	0.917	–0.103	0.051	–0.255	–0.162
K	0.559	0.51	0.519	–0.209	0.182
Ti	0.815	0.23	0.411	–0.056	–0.2
Zr	0.845	0.056	0.246	–0.304	0.261
Y	0.953	–0.094	–0.143	–0.155	0.097
Ba	0.647	0.669	0.061	0.004	–0.08
Eigenvalues	14.281	3.723	3.157	1.937	1.286
% of Total Variance	52.893	13.79	11.693	7.174	4.765
Cumulative %	52.893	66.682	78.375	85.549	90.314

## References

[1] Dalu T., Wasserman R.J., Tonkin J.D., Mwedzi T., Magoro M.L., Weyl O.L.F. (2017). Water or sediment? Partitioning the role of water column and sediment chemistry as drivers of macroinvertebrate communities in an austral south african stream. Sci. Total Environ..

[2] Sarkar S.K., Mondal P., Biswas J.K., Kwon E.E., Ok Y.S., Rinklebe J. (2017). Trace elements in surface sediments of the hooghly (ganges) estuary: Distribution and contamination risk assessment. Environ. Geochem. Health.

[3] Ranjbar Jafarabadi A., Riyahi Bakhtiyari A., Shadmehri Toosi A., Jadot C. (2017). Spatial distribution, ecological and health risk assessment of heavy metals in marine surface sediments and coastal seawaters of fringing coral reefs of the persian gulf, iran. Chemosphere.

[4] Pinto M.M.S.C., Silva E.A.F., Silva M.M.V.G., Melo-Gonçalves P., Candeias C. (2014). Environmental risk assessment based on high-resolution spatial maps of potentially toxic elements sampled on stream sediments of santiago, cape verde. Geosciences.

[5] Cabral Pinto M.M.S., Ferreira da Silva E.A. (2018). Heavy metals of santiago island (cape verde) alluvial deposits: Baseline value maps and human health risk assessment. Int. J. Environ. Res. Public Health.

[6] Sharifinia M., Taherizadeh M., Namin J.I., Kamrani E. (2018). Ecological risk assessment of trace metals in the surface sediments of the persian gulf and gulf of oman: Evidence from subtropical estuaries of the iranian coastal waters. Chemosphere.

[7] Gedik K. (2018). Bioaccessibility of heavy metals in rapa whelk rapana venosa valenciennes 1846 assessing human health risk using an in vitro digestion model. Hum. Ecol. Risk Assess..

[8] Peng J.-F., Song Y.-H., Yuan P., Cui X.-Y., Qiu G.-L. (2009). The remediation of heavy metals contaminated sediment. J. Hazard. Mater..

[9] Fu J., Hu X., Tao X., Yu H., Zhang X. (2013). Risk and toxicity assessments of heavy metals in sediments and fishes from the yangtze river and taihu lake, china. Chemosphere.

[10] Zhang Z.W., Xu X.R., Sun Y.X., Yu S., Chen Y.S., Peng J.X. (2014). Heavy metal and organic contaminants in mangrove ecosystems of china: A review. Environ. Sci. Pollut. Res..

[11] Guan Q., Wang L., Pan B., Guan W., Sun X., Cai A. (2016). Distribution features and controls of heavy metals in surface sediments from the riverbed of the ningxia-inner mongolian reaches, yellow river, china. Chemosphere.

[12] Chen L., Zhou S., Shi Y., Wang C., Li B., Li Y., Wu S. (2018). Heavy metals in food crops, soil, and water in the lihe river watershed of the taihu region and their potential health risks when ingested. Sci. Total Environ..

[13] Yi Y., Yang Z., Zhang S. (2011). Ecological risk assessment of heavy metals in sediment and human health risk assessment of heavy metals in fishes in the middle and lower reaches of the yangtze river basin. Environ. Pollut..

[14] Wang Y.B., Liu C.W., Wang S.W. (2015). Characterization of heavy-metal-contaminated sediment by using unsupervised multivariate techniques and health risk assessment. Ecotoxicol. Environ. Saf..

[15] Tarafdar A., Sinha A. (2017). Cancer risk assessment of polycyclic aromatic hydrocarbons in the soils and sediments of india: A meta-analysis. Environ. Manag..

[16] Tepanosyan G., Sahakyan L., Belyaeva O., Maghakyan N., Saghatelyan A. (2017). Human health risk assessment and riskiest heavy metal origin identification in urban soils of yerevan, armenia. Chemosphere.

[17] Leung H.M., Leung A.O., Wang H.S., Ma K.K., Liang Y., Ho K.C., Cheung K.C., Tohidi F., Yung K.K. (2014). Assessment of heavy metals/metalloid (as, pb, cd, ni, zn, cr, cu, mn) concentrations in edible fish species tissue in the pearl river delta (prd), china. Mar. Pollut. Bull..

[18] Qi J., Zhang H., Li X., Lu J., Zhang G. (2016). Concentrations, spatial distribution, and risk assessment of soil heavy metals in a zn-pb mine district in southern china. Environ. Monit. Assess..

[19] Lecrivain N., Aurenche V., Cottin N., Frossard V., Clement B. (2018). Multi-contamination (heavy metals, polychlorinated biphenyls and polycyclic aromatic hydrocarbons) of littoral sediments and the associated ecological risk assessment in a large lake in france (lake bourget). Sci. Total Environ..

[20] Szefer P. (1998). Distribution and behaviour of selected heavy metals and other elements in various components of the southern baltic ecosystem. Appl. Geochem..

[21] Yang Z., Wang Y., Shen Z., Niu J., Tang Z. (2009). Distribution and speciation of heavy metals in sediments from the mainstream, tributaries, and lakes of the yangtze river catchment of wuhan, china. J. Hazard. Mater..

[22] Chai L., Wang Z., Wang Y., Yang Z., Wang H., Wu X. (2010). Ingestion risks of metals in groundwater based on tin model and dose-response assessment—A case study in the xiangjiang watershed, central-south china. Sci. Total Environ..

[23] Gao X., Chen C.T. (2012). Heavy metal pollution status in surface sediments of the coastal bohai bay. Water Res..

[24] Mao L., Mo D., Guo Y., Fu Q., Yang J., Jia Y. (2013). Multivariate analysis of heavy metals in surface sediments from lower;reaches of the xiangjiang river, southern china. Environ. Earth Sci..

[25] Zhang Y., Chu C., Li T., Xu S., Liu L., Ju M. (2017). A water quality management strategy for regionally protected water through health risk assessment and spatial distribution of heavy metal pollution in 3 marine reserves. Sci. Total Environ..

[26] Ogunlaja A., Ogunlaja O.O., Okewole D.M., Morenikeji O.A. (2019). Risk assessment and source identification of heavy metal contamination by multivariate and hazard index analyses of a pipeline vandalised area in lagos state, nigeria. Sci. Total Environ..

[27] Chen C.F., Ju Y.R., Chen C.W., Dong C.D. (2016). Vertical profile, contamination assessment, and source apportionment of heavy metals in sediment cores of kaohsiung harbor, taiwan. Chemosphere.

[28] Pandey L.K., Park J., Son D.H., Kim W., Islam M.S., Choi S., Lee H., Han T. (2019). Assessment of metal contamination in water and sediments from major rivers in south korea from 2008 to 2015. Sci. Total Environ..

[29] Cabral Pinto M.M.S., Silva M.M.V., Ferreira da Silva E.A., Marinho-Reis A.P. (2017). The cancer and non-cancer risk of santiago island (cape verde) population due to potential toxic elements exposure from soils. Geosciences.

[30] Cabral Pinto M.M.S., Marinho-Reis A.P., Almeida A., Ordens C.M., Silva M.M.V.G., Freitas S., Simões M.R., Moreira P.I., Dinis P.A., Diniz M.L. (2018). Human predisposition to cognitive impairment and its relation with environmental exposure to potentially toxic elements. Environ. Geochem. Health.

[31] Cabral Pinto M.M.S., Marinho-Reis P., Almeida A., Pinto E., Neves O., Inácio M., Gerardo B., Freitas S., Simões M.R., Dinis P.A. (2019). Links between cognitive status and trace element levels in hair for an environmentally exposed population: A case study in the surroundings of the estarreja industrial area. Int. J. Environ. Res. Public Health.

[32] Cabral Pinto M.M.S., Ordens C.M., Condesso de Melo M.T., Inácio M., Almeida A., Pinto E., Ferreira da Silva E.A. (2019). An inter-disciplinary approach to evaluate human health risks due to long-term exposure to contaminated groundwater near a chemical complex. Expo. Health.

[33] Cabral-Pinto M.M., Inácio M., Neves O., Almeida A.A., Pinto E., Oliveiros B., da Silva E.A.F. (2019). Human health risk assessment due to agricultural activities and crop consumption in the surroundings of an industrial area. Expo. Health.

[34] Zhang L., Li J. (1987). Chatacteristics of the xiangjiang river system. Acta Geogr. Sin..

[35] Zhang S., Dong W., Zhang L., Chen X. (1989). Geochemical characteristics of heavy metals in the xiangjiang river, china. Hydrobiologia.

[36] Dong W., Zhang L., Zhang S. (1992). The reseatch on the distribution and forms of heavy metals in the xiangjiang river sediments. Chin. GeoGraph. Sci..

[37] Wang Y., Yang Z., Peng B., Chai L., Wu B., Wu R. (2013). Biotreatment of chromite ore processing residue bypannonibacter phragmitetus bb. Environ. Sci. Pollut. Res..

[38] Lin C., He M., Liu X., Wei G., Liu S. (2013). Contamination and ecological risk assessment of toxic trace elements in the xi river, an urban river of shenyang city, china. Environ. Monit. Assess..

[39] Liao Y., Min X., Yang Z., Chai L., Zhang S., Wang Y. (2014). Physicochemical and biological quality of soil in hexavalent chromium-contaminated soils as affected by chemical and microbial remediation. Environ. Sci. Pollut. Res..

[40] Szefer P., Kusak A., Szefer K., Jankowska H., WoŁowicz M., Ali A.A. (1995). Distribution of selected metals in sediment cores of puck bay, baltic sea. Mar. Pollut. Bull..

[41] Chai L., Li H., Yang Z., Min X., Liao Q., Liu Y., Men S., Yan Y., Xu J. (2017). Heavy metals and metalloids in the surface sediments of the xiangjiang river, hunan, china: Distribution, contamination, and ecological risk assessment. Environ. Sci. Pollut. Res. Int..

[42] Lee A., Willcox B. (2014). Minkowski generalizations of ward’s method in hierarchical clustering. J. Classif..

[43] USEPA (2004). Risk Assessment Guidance for Superfund Volume I: Human Health Evaluation Manual (Part E, Supplemental Guidance for Dermal Risk Assessment).

[44] USEPA (2002). Supplemental Guidance for Developing Soil Screening Levels for Superfund Sites.

[45] USEPA (2011). Exposure Factors Handbook 2011 Edition (Final).

[46] USDE (2013). The Risk Assessment Information System (Rais).

[47] Simmonds V., Jahangiryar F., Moazzen M., Ravaghi A. (2017). Distribution of base metals and the related elements in the stream-sediments around the ahar area (nw iran) and their implications. Chem. Erde.

[48] Martin R.F., Wülser P. (2014). Niobium and tantalum in minerals: Siderophile, chalcophile or lithophile, and polyvalent. J. Geochem. Expo..

[49] Holzheid A., Palme H. (2007). The formation of eucrites: Constraints from metal-silicate partition coefficients. Meteorit. Planet. Sci..

[50] Mao L., Mo D., Yang J., Jia Y., Guo Y. (2013). Concentration and pollution assessment of hazardous metal elements in sediments of the xiangjiang river, china. J. Radioanal. Nucl. Chem..

[51] Mao L., Mo D., Yang J., Guo Y., Lv H. (2014). Rare earth elements geochemistry in surface floodplain sediments from the xiangjiang river, middle reach of changjiang river, china. Quat. Int..

[52] Anand R.R., Gilkes R.J. (1987). Variations in the properties of iron oxides within individual specimens of lateritic duricrust. Soil Res..

[53] Trolard F., Bourrie G., Jeanroy E., Herbillon A.J., Martin H. (1995). Trace metals in natural iron oxides from laterites: A study using selective kinetic extraction. Geochim. Cosmochim. Acta.

[54] Soubrand-Colin M., Neel C., Bril H., Grosbois C., Caner L. (2007). Geochemical behaviour of ni, cr, cu, zn and pb in an andosol–cambisol climosequence on basaltic rocks in the french massif central. Geoderma.

[55] Li F., Qiu Z., Zhang J., Liu W., Liu C., Zeng G. (2017). Investigation, pollution mapping and simulative leakage health risk assessment for heavy metals and metalloids in groundwater from a typical brownfield, middle china. Int. J. Environ. Res. Public Health.

[56] Liu J., Xu Y., Cheng Y., Zhao Y., Pan Y., Fu G., Dai Y. (2017). Occurrence and risk assessment of heavy metals in sediments of the xiangjiang river, china. Environ. Sci. Pollut. Res. Int..

[57] Rubio B., Nombela M.A., Vilas F. (2000). Geochemistry of major and trace elements in sediments of the ria de vigo (nw spain): An assessment of metal pollution. Mar. Pollut. Bull..

[58] Wu H., Zeng G., Jie L., Zhang J., Cai Q., Lu H., Li X., Zhu H., Hu C., Sheng S. (2013). Changes of soil microbial biomass and bacterial community structure in dongting lake: Impacts of 50,000 dams of yangtze river. Ecol. Eng..

[59] Freret-Meurer N.V., Andreata J.V., Meurer B.C., Manzano F.V., Baptista M.G.S., Teixeira D.E., Longo M.M. (2010). Spatial distribution of metals in sediments of the ribeira bay, angra dos reis, rio de janeiro, brazil. Mar. Pollut. Bull..

[60] Zeng X., Liu Y., You S., Zeng G., Tan X., Hu X., Hu X., Huang L., Li F. (2015). Spatial distribution, health risk assessment and statistical source identification of the trace elements in surface water from the xiangjiang river, china. Environ. Sci. Pollut. Res. Int..

[61] Pan L., Fang G., Wang Y., Wang L., Su B., Li D., Xiang B. (2018). Potentially toxic element pollution levels and risk assessment of soils and sediments in the upstream river, miyun reservoir, china. Int. J. Environ. Res. Public Health.

